# Assessment of vascular invasion of pancreatic ductal adenocarcinoma based on CE-boost black blood CT technique

**DOI:** 10.1186/s13244-024-01870-x

**Published:** 2024-12-05

**Authors:** Yue Lin, Tongxi Liu, Yingying Hu, Yinghao Xu, Jian Wang, Sijia Guo, Sheng Xie, Hongliang Sun

**Affiliations:** 1 Department of Radiology, China–Japan Friendship Hospital, Beijing, China; 2 CT Clinical Research Department, CT Business Unit, Canon Medical Systems (China) Co., Ltd., Beijing, China; 3 Peking University China–Japan Friendship School of Clinical Medicine, Beijing, China

**Keywords:** Deep learning reconstruction, Hybrid-iterative reconstruction, Advanced intelligent clear-IQ engine, Black blood CT technique, Pancreatic ductal adenocarcinoma

## Abstract

**Objectives:**

To explore the diagnostic efficacy of advanced intelligent clear-IQ engine (AiCE) and adaptive iterative dose reduction 3D (AIDR 3D), combination with and without the black blood CT technique (BBCT), for detecting vascular invasion in patients diagnosed with nonmetastatic pancreatic ductal adenocarcinoma (PDAC).

**Methods:**

A total of 35 consecutive patients diagnosed with PDAC, proceeding with contrast-enhanced abdominal CT scans, were enrolled in this study. The arterial and portal venous phase images were reconstructed using AiCE and AIDR 3D. The corresponding BBCT images were established as AiCE–BBCT and AIDR 3D–BBCT, respectively. Two observers scored the image quality independently. Cohen’s kappa (k) value or intraclass correlation coefficient (ICC) was used to analyze consistency. The diagnostic performance of four algorithms in detecting vascular invasion in PDAC patients was assessed using the area under the curve (AUC).

**Results:**

The AiCE and AiCE–BBCT groups demonstrated superior image noise and diagnostic acceptability compared with AIDR 3D and AIDR 3D–BBCT groups (all *p* < 0.001), and the k value was 0.861–0.967 for both reviewers. In terms of diagnostic capability for vascular invasion in PDAC, the AiCE–BBCT group exhibited higher specificity (95.0%) and sensitivity (93.3%) compared to the AIDR 3D and AIDR 3D–BBCT groups, with an AUC of 0.942 (95% CI: 0.849–1.000, *p* < 0.05). Furthermore, all vascular evaluations conducted using AiCE–BBCT demonstrated better consistency (ICC: 0.847–0.935).

**Conclusion:**

The BBCT technique in conjunction with AiCE could lead to notable enhancements in both the image quality of PDAC images and the diagnostic performance for tumor vascular invasion.

**Critical relevance statement:**

Better diagnostic accuracy of vascular invasion of PDAC based on BBCT in combination with an AiCE is a critical factor in determining treatment strategies and patient outcomes.

**Key Points:**

- Identifying vascular invasion of PDAC is important for prognostication.
- Combined images provide improved image quality and higher diagnostic accuracy.
- Combined images can excellently display the vascular wall and invasion.

**Graphical Abstract:**

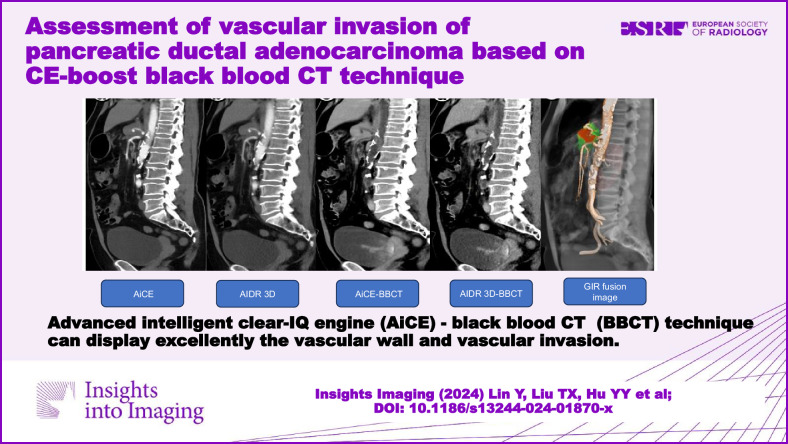

## Introduction

Pancreatic ductal adenocarcinoma (PDAC) [1–3] is a highly lethal malignant tumor, with surgical resection being the only potentially curative option. By undergoing surgery, the 5-year survival rate can increase to 12–20%, compared to less than 1% for cases that are deemed unresectable. Unfortunately, only a small fraction of patients (less than 20%) are eligible for surgical resection, while the majority present with involvement of major abdominal vessels such as the inferior vena cava and aorta, as well as distant metastasis [4, 5]. In cases where distant metastases are absent, the presence of vascular invasion holds significant clinical importance as it serves as a prognostic indicator and impacts the overall survival of PDAC patients. Common sites of tumor invasion include the celiac artery (CA), superior mesenteric artery (SMA), common hepatic artery (CHA), superior mesenteric vein (SMV), and portal vein (PV). Vascular invasion also plays a crucial role in determining suitable treatment options. PDAC is classified into resectable, borderline resectable, and unresectable categories based on the relationship between tumors and vascular structure by National Comprehensive Cancer Network (NCCN) guidelines [6]. However, vascular invasion often requires surgical or pathological confirmation, which can lead to obvious surgical impairment in patients with unresectable PDAC. Accordingly, the accurate diagnosis of vascular invasion in PDAC patients holds great significance as it enables surgeons to make informed decisions regarding surgical procedures and provides valuable prognostic information [7, 8].

Computed tomography (CT) [9] is one of the primary examination approaches used for assessing vascular and structural invasion in lesions, as well as detecting distant metastases. It is widely regarded as the most effective and commonly employed method in clinical practice. CT has demonstrated accuracies of 77% in predicting resectability and 93% in predicting unresectability of PDAC cases [4, 7, 10]. In cases where distant metastasis is not present, the extent of vascular invasion observed on CT scans is considered the most crucial factor for predicting resectability and determining the prognosis of PDAC patients.

To address image noise and enhance image quality, researchers have developed various reconstruction algorithms and post-processing techniques [11, 12]. One such technique is deep learning reconstruction (DLR), which utilizes a deep convolutional neural network. To our knowledge, there are currently two commercially available CT image reconstruction algorithms using DL methods cleared by the FDA, TrueFidelity by GE Healthcare and advanced intelligent clear-IQ engine (AiCE) by Canon Medical Systems. Both TrueFidelity and AiCE were rated superior for subjective image quality by radiologists [13]. The purpose of this study is to explore the diagnostic capability of AiCE, an example of a commercially available DLR tool designed for CT imaging [14]. It leverages the power of deep learning to differentiate true signals from noise within an image [15]. A previous study [16] demonstrated that AiCE, an example of a commercially available DLR tool, exhibits superior image quality characteristics compared to model-based iterative reconstruction or filtered back projection techniques in abdominal CT images. Nevertheless, in routine clinical applications, hybrid-iterative reconstruction (hybrid-IR) methods such as adaptive iterative dose reduction 3D (AIDR 3D) are commonly employed. Meanwhile, noise-reducing reconstruction algorithms such as AIDR 3D act as a noise-reduction technique. Therefore, it is essential to compare the clinical utility of DLR (AiCE) with hybrid-IR (AIDR 3D). A separate study [15] indicated that DLR (AiCE) exhibits superior image noise and quality in coronary computed tomography angiography when compared to AIDR 3D. Moreover, the use of AiCE has demonstrated the ability to reduce radiation dose without compromising image quality when compared with AIDR 3D [11, 17]. These findings highlight the potential benefits of utilizing AiCE in clinical practice for image quality during lower-dose abdominal CT examinations.

Like the contrast-enhancement boost (CE-boost) technology [18, 19], the black blood CT technique (BBCT) utilizes subtraction technology and registration algorithms. In BBCT, we subtracted the arterial image from the delayed phase image and the portal venous phase image from the pre-contrast phase image. These images after subtraction are then overlaid to the initial delayed phase or pre-contrast phase image. This process can effectively reduce lumen brightness and enhance the visualization of the vascular wall. Previous research [20] has demonstrated that BBCT can provide clear visualization of the carotid artery wall, precise diagnosis of plaque burden, and stenosis rate in the carotid artery, resulting in a significant improvement in image quality. Given that the vascular wall exhibits delayed enhancement, BBCT allows for the distinction between the lumen and vascular wall structure of abdominal vessels by overlying the delayed-phase images. BBCT enhances the display of the vascular wall which is valuable for evaluating vascular invasion in PDAC.

AiCE is a new deep learning image reconstruction algorithm, and AIDR 3D is a hybrid iterative reconstruction algorithm, they are two different reconstruction algorithms that can be obtained simultaneously during patient scanning. Based on this background, we hypothesized that combining the BBCT technique with AiCE could yield optimal image quality in PDAC, particularly for assessing tumor vascular invasion. Therefore, the objective of the present study was to explore different iterative reconstruction algorithms (AiCE and AIDR 3D) in conjunction with and without the BBCT technology. Our aim was to significantly improve the diagnostic performance for evaluating vascular invasion in patients with nonmetastatic PDAC.

## Material and methods

### Study population

This prospective study was approved by the Institutional Review Board of our hospital, and informed consent was obtained from all patients.

**Fig. 1:**
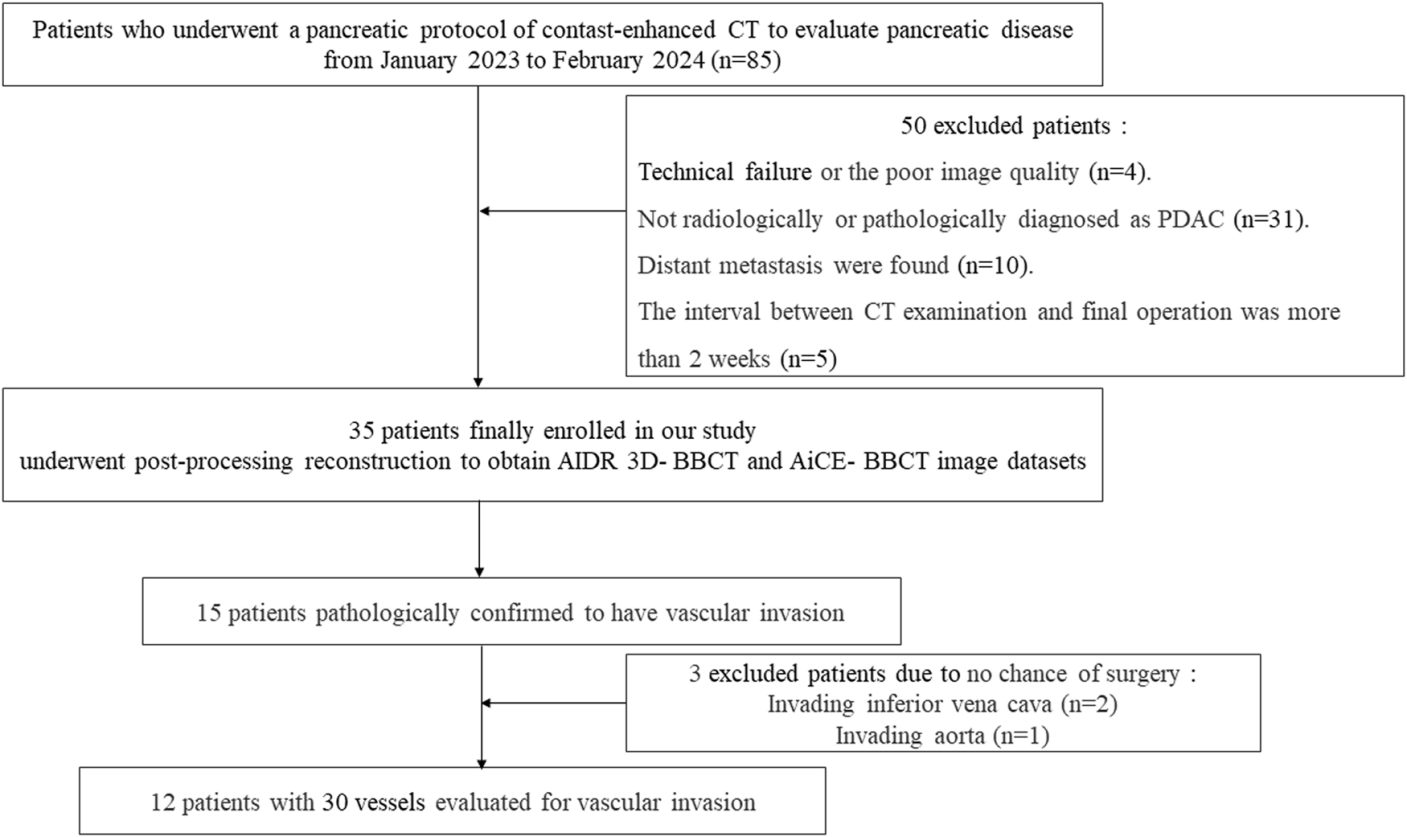
A flow chart of subject enrollment and study design. Based on the predefined exclusion criteria, 35 patients successfully enrolled in this study

Between January 2023 and February 2024, we enrolled 85 consecutive patients with clinically diagnosed pancreatic disease and obstructive jaundice, conditions highly suggestive of pancreatic cancer, who proceeded with abdominal enhanced CT for the evaluation of PDAC in this study. The exclusion criteria were: (a) technical failures or poor image quality, (b) not receiving a radiological or pathological diagnosis of PDAC, (c) distant metastasis confirmed by clinical or imaging examination, and (d) more than two weeks’ interval between the CT examination and the final operation. Finally, a total of 35 eligible patients were enrolled in this study. We evaluated 175 vessels of 35 enrolled PDAC patients for vascular invasion, according to the criteria used to determine vessel invasion: (1) circumferential tumor contact, (2) vascular caliber thinning, or (3) contour irregularity, and 30 vessels, not major abdominal vessels, with 12 patients were found to have vascular invasion imaging findings. Further, we performed a more detailed vascular evaluation of the 30 vessels involved (Fig. 1).

### CT scan and image post-processing

All patients underwent pancreatic protocol CT using a 320-row CT scanner (Aquilion ONE GENESIS Edition, Canon Medical System Corporation, Japan). The scanning protocol included four phases: pre-contrast, arterial, portal venous, and delayed phases. The following scan parameters were used: rotation time of 0.5 s, tube voltage of 100 kVp, automatic exposure control with a noise index of 7.5 HU (using ^SURE^ Exposure 3D), and a detector configuration of 80 × 0.5 mm with a pitch standard of 0.813/65.0. The reconstructed slice thickness and increment were both set to 1 mm, with a matrix size of 512 × 512 and a field of view of 500 mm.

After acquiring a non-enhanced scan of the abdomen, a nonionic contrast agent and normal saline (NS) were injected through the median cubital vein at a rate of 4.0 mgI/s using a double-barreled high-pressure injection syringe. Bolus tracking technology was employed to initiate the collection of the arterial phase 10 s after the aortic CT value reached 200 HU. The portal venous phase began 25 s after the arterial phase, and the delayed phase commenced 60 s after the portal venous phase.

Immediately after the scanning, AiCE, and AIDR 3D image datasets were automatically generated. Additionally, BBCT images were obtained using dedicated analysis software installed in the CT workstation (Aquilion ONE, Canon Medical Systems). The BBCT process involved subtracting the arterial and portal venous phases from the delayed and pre-contrast phases, respectively. The resulting images are subsequently superimposed onto the initial delayed or pre-contrast phase image. This method effectively diminishes lumen brightness and enhances the visualization of the vascular wall [18]. The reconstructed images for each phase (pre-contrast, arterial, portal venous, and delayed) were separately generated using both AIDR 3D and AiCE, with a slice thickness of 1 mm and a 1 mm interval. BBCT images were created using the dedicated analysis software, which performed the subtraction process described above. We ultimately can obtain 8 data subsets for each case that resulted from the reconstruction and subtraction analysis process, including the arterial and venous phases of the AiCE, AiCE–BBCT, AIDR 3D–BBCT, and AIDR 3D groups.

### Qualitative image quality evaluation

Two radiologists randomly evaluated the arterial and portal phase data sets, knowing nothing about the pathological findings or clinical information. All observed images have the same position and layer thickness settings. The radiologists evaluated the image quality of arterial and portal venous datasets. The image quality, including image noise and diagnostic acceptability, was independently scored by two radiologists, using a 5-point scale: 1, unacceptable; 2, below average; 3, average; 4, above average; and 5, excellent [21]. Prior to image quality evaluation, the two radiologists underwent a training session including abdominal CT images from 10 patients to get familiar with the scoring system [17].

### Diagnostic performance evaluation

The observers in this study assessed the invasion of five vessels for each patient: the CA, SMA, CHA, SMV, and PV. The criteria used to determine vessel invasion were as follows: (1) circumferential tumor contact, (2) vascular caliber thinning, or (3) contour irregularity. The results of tumor-vessel contact were confirmed by intraoperative records and final pathology results.

A total of 35 enrolled PDAC patients were evaluated. Each patient has evaluated eight sets of images, including arterial and portal venous phases of the four reconstruction algorithms (AiCE, AiCE–BBCT, AIDR 3D–BBCT, and AIDR 3D), meaning that each reviewer evaluated 280 sets of images in this study.

The surgical procedures performed were categorized as exploratory laparotomies, pancreatoduodenectomies, or partial pancreatectomies.

### Vascular evaluation

According to the criteria used to diagnose vessel invasion, PDAC patients without vascular invasion were excluded from the vascular evaluation. Meanwhile, two patients with involvement of major abdominal vessels (the inferior vena cava and the aorta) were not included in the analysis, as they were inoperable cases according to the NCCN criteria for PDAC staging [6]. Finally, 30 vessels, not major abdominal vessels, with 12 patients out of 175 vessels with 35 enrolled PDAC patients were found to have vascular invasion imaging findings. Among these vessels, there were 12 cases of CA involvement, 11 cases of SMA involvement, 5 cases of CHA involvement, and 2 cases of SMV involvement. Further, we performed a more detailed vascular evaluation of the 30 vessels involved.

To evaluate the accuracy of different reconstruction methods, we compared the CT images with the pathological findings or results from exploratory laparotomies. Specifically, we analyzed the extent of tumor contact with the vessel circumference (> 180 or ≤ 180), the presence of unilateral or bilateral stenosis, the regularity or irregularity of the vessel lumen, and the length of tumor invasion into the blood vessel (> 1.2 cm or ≤ 1.2 cm) under different reconstruction methods. The results obtained using AiCE–BBCT by two radiologists were known as AiCE–BBCTR1 and AiCE–BBCTR2.

### Radiation dose

To estimate the effective radiation dose (mSv), the dose-length product (DLP; mGy·cm) was multiplied by a conversion factor of 0.015 (mSv·mGy^−^^1^·cm^−1^). This conversion factor is commonly used to calculate the effective dose from DLP in CT imaging. The four reconstruction methods in this study were all compared under the premise of the same scan and the same radiation dose, and the radiation dose was similar to the radiation dose of an abdominal enhanced scan in other studies, which also laid the foundation for future exploration of low radiation dose scanning schemes [16].

### Statistical analysis

All statistical process was performed in IBM SPSS 25.0 software. Numerical variables were presented as mean ± standard deviation (SD). Two-way random model intraclass correlation coefficient (ICC) and Cohen’s kappa (k) statistic were used to assess the inter-reader agreement. The image quality was independently scored by two radiologists, using a 5-point scale. The k value was used to assess the consistency of the inter-observer image quality score. The ICC was used to assess the consistency of the inter-observer vascular evaluation, including tumor contact of the vessel circumference (> 180 or ≤ 180), unilateral or bilateral stenosis, regular or irregular lumen, tumor invasion of blood vessel involvement length (> 1.2 cm or ≤ 1.2 cm). Interpretation of ICC values is as follows: < 0.50 stands for poor consistency, 0.50–0.75 stands for moderate consistency, 0.76–0.90 stands for good consistency, and > 0.90 stands for excellent consistency [21]. Interpretation of k values is as follows: ≤ 0.40 stands for poor consistency, 0.41–0.80 stands for good consistency, and ≥ 0.81 stands for excellent consistency [21]. Additionally, the diagnostic accuracy of the four reconstruction methods (AiCE, AiCE–BBCT, AIDR 3D–BBCT, and AIDR 3D) for vascular invasion in patients with PDAC was evaluated using receiver operating characteristic curve, and the area under the curve (AUC) value. The DeLong test was used to compare the AUCs between these algorithms. A *p*-value < 0.05 was considered statistically significant.

## Results

### Study population

Finally, a total of 35 patients with PDAC were enrolled in the analysis. In our study, there were 20 men and 15 women, with a mean age of 66.13 ± 11.01 years (range: 46–89 years). The mean tumor size was 2.83 ± 0.92 cm (range: 1.3–5.9 cm) among the included patients. Out of the 35 PDAC cases, 24 were located in the pancreatic head or uncinate process, while the remaining 11 cases were located in the pancreatic body or tail. Regarding the type of operation performed, 9 patients underwent pancreatoduodenectomy, 21 patients underwent partial pancreatectomy, and 5 patients underwent exploratory laparotomy.

### Radiation dose

In our research, for the nonenhanced phase, arterial phase, portal venous phase, and delayed phase, the effective radiation dose was 2.96 ± 1.41 mSv, 5.91 ± 2.82 mSv, 5.91 ± 2.82 mSv, and 5.91 ± 2.82 mSv, respectively. The radiation dose was similar to the radiation dose of abdominal-enhanced scans in other studies [16].

### Qualitative image quality evaluation

**Fig. 2:**
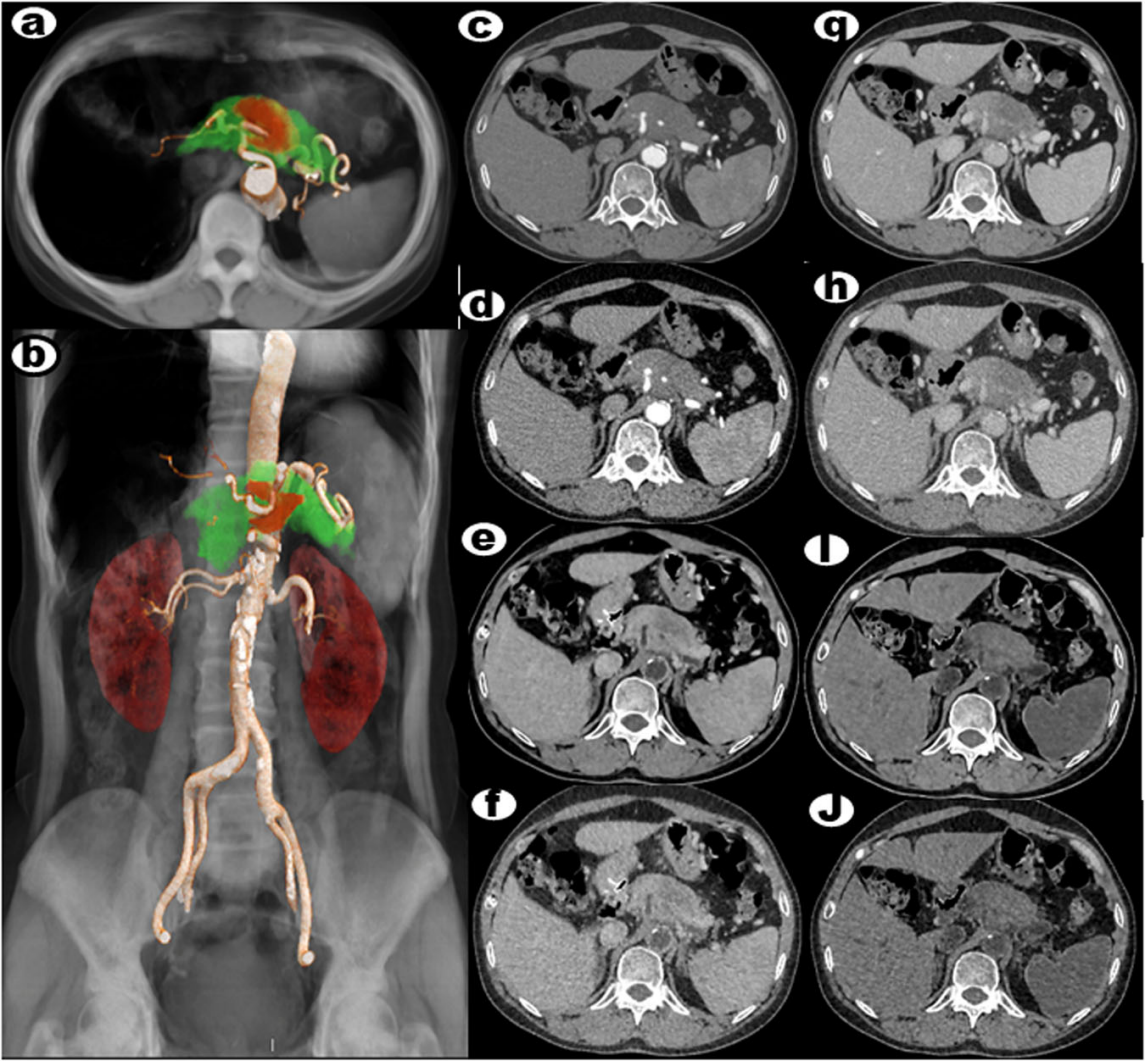
A 56-year-old woman presented with PDAC located in the body region of the pancreas. **a**, **b** Global illumination rendering (GIR) fusion images reconstructed using the AiCE algorithm were used to visualize the tumor invasion. In these fusion images, normal pancreatic parenchyma was displayed in green, while the tumor was depicted in red. **c**–**f** Axial CT images at the arterial phase were reconstructed using AiCE, AIDR 3D, AiCE–BBCT, and AIDR 3D–BBCT algorithms, revealing the presence of a pancreatic tumor (indicated by an arrow). **g**–**j** Similarly, axial CT images at the portal venous phase, reconstructed using the same algorithms, confirmed the presence of the tumor (also indicated by an arrow). The image reconstruction utilized a slice thickness of 1 mm. These imaging findings provide a visual representation of the PDAC and demonstrate the application of various algorithms, including AiCE, AIDR 3D, AiCE–BBCT, and AIDR 3D–BBCT, in detecting and assessing the tumor at different phases of contrast enhancement

Table 1 summarizes the results for image noise, diagnostic acceptability, and k value. The subjective image analysis of the AIDR 3D, AIDR 3D–BBCT, AiCE, and AiCE–BBCT in the arterial and portal venous phases by the two reviewers had significant statistical differences (all *p* < 0.01). In the AiCE and AiCE–BBCT groups, both arterial and portal venous reconstructed images exhibited superior image noise and diagnostic acceptability compared to the AIDR 3D–BBCT and AIDR 3D groups (all *p* < 0.001 for both reviewers). We compared the image noise and diagnostic acceptability between the AIDR 3D and AIDR 3D–BBCT groups, and the AiCE and AiCE–BBCT groups, in either arterial or portal venous phases, but it was not statistically significant. This case shows that AiCE, AiCE–BBCT, AIDR 3D–BBCT, and AIDR 3D groups in either arterial or portal venous reconstructed images of a PDAC patient (Fig. 2). The calculated k value ranged from 0.861 to 0.967, representing a perfect consistency between the two observers.

**Table 1:** Subjective image analysis of the AIDR 3D, AIDR 3D–BBCT, AiCE, and AiCE–BBCT in the arterial and portal venous phases

Parameter	AIDR 3D			AIDR 3D–BBCT			AiCE			AiCE–BBCT		
	Reviewer 1	Reviewer 2	ĸ value	Reviewer 1	Reviewer 2	ĸ value	Reviewer 1	Reviewer 2	ĸ value	Reviewer 1	Reviewer 2	ĸ value
Arterial phase												
Image noise	3.31 ± 0.72 (2–4)	3.42 ± 0.71 (2–4)	0.994	3.52 ± 0.83 (2–4)	3.51 ± 0.83 (2–4)	0.941	4.21 ± 0.52 (4–5)	4.23 ± 0.49 (4–5)	0.955	4.31 ± 0.51 (4–5)	4.31 ± 0.44 (4–5)	0.967
Diagnostic acceptability	3.31 ± 0.81 (2–4)	3.32 ± 0.82 (2–4)	0.860	3.37 ± 0.71 (2–4)	3.32 ± 0.81 (2–4)	0.885	4.31 ± 0.51 (4–5)	4.29 ± 0.51 (4–5)	0.928	4.33 ± 0.52 (4–5)	4.32 ± 0.48 (4–5)	0.934
Portal venous phase												
Image noise	3.22 ± 0.82 (2–4)	3.21 ± 0.87 (2–4)	0.839	3.41 ± 0.81 (2–4)	3.22 ± 0.89 (2–4)	0.920	4.25 ± 0.66 (3–5)	4.19 ± 0.58 (3–5)	0.901	4.21 ± 0.61 (3–5)	3.93 ± 0.61 (3–5)	0.867
Diagnostic acceptability	3.21 ± 0.81 (2–4)	3.30 ± 0.74 (2–4)	0.944	3.34 ± 0.80 (2–4)	3.31 ± 0.64 (2–4)	0.868	4.21 ± 0.62 (3–5)	4.18 ± 0.61 (3–5)	0.861	4.14 ± 0.62 (3–5)	4.01 ± 0.61 (3–5)	0.861

	Arterial phase				Portal venous phase			
	AIDR 3D	AIDR 3D–BBCT	AiCE	AiCE–BBCT	AIDR 3D	AIDR 3D–BBCT	AiCE	AiCE–BBCT
AIDR 3D	**/**	NS	< 0.001	< 0.001	**/**	NS	< 0.001	< 0.001
AIDR 3D–BBCT		**/**	< 0.001	< 0.001		**/**	< 0.001	< 0.001
AiCE–BBCT			NS	/			NS	**/**

Data are means ± SDs, with interquartile ranges in parentheses. The k value was used to analyze consistency

*NS* no significant differences

### Diagnostic performance evaluation

**Fig. 3:**
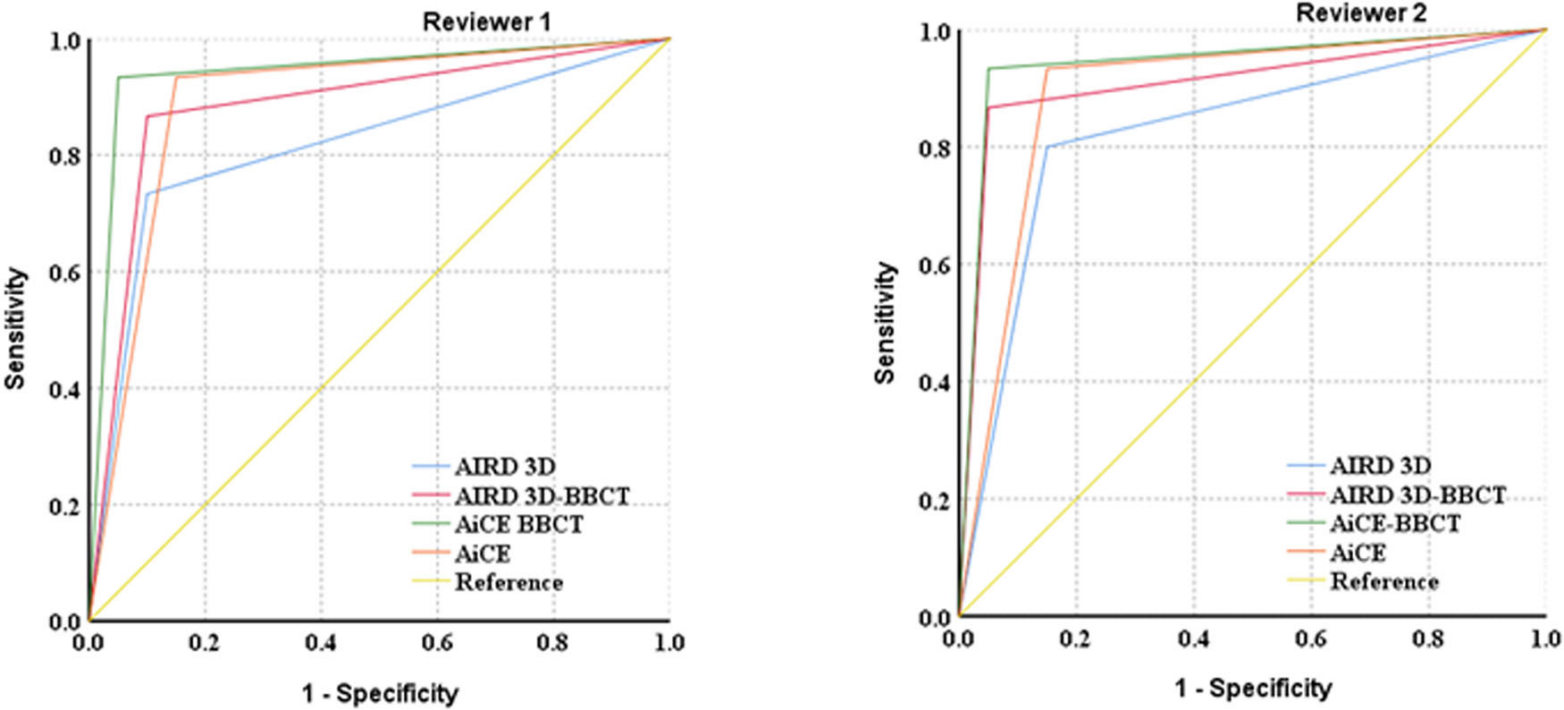
The diagnostic performance of the AIDR 3D, AIDR 3D–BBCT, AiCE, and AiCE- BBCT for vascular invasion in PDAC with two reviewers, using pathological findings or results from exploratory laparotomies as the gold standard

Finally, we analyzed 35 patients with 175 vessels of diagnostic performance, with pathological findings or results from exploratory laparotomies serving as the gold standard (Table 2). For both reviewers, the diagnostic capability for vascular invasion in PDAC, the AiCE–BBCT group exhibited higher specificity (95.0%) and sensitivity (93.3%) compared to the AIDR 3D and AIDR 3D–BBCT groups, with an AUC of 0.942 (95% CI: 0.849–1.000) (Fig. 3). AiCE–BBCT group significantly improved the accuracy compared with AIDR 3D and AIDR 3D–BBCT groups for 2 readers (*p* < 0.05), while there were no significant differences for AiCE and AiCE–BBCT groups for 2 readers.

**Table 2:** The diagnostic performance of the AIDR 3D, AIDR 3D–BBCT, AiCE, and AiCE–BBCT for vascular invasion in PDAC with two reviewers

Parameter	AIDR 3D			AIDR3D–BBCT			AiCE			AiCE–BBCT		
	Sensitivity	Specificity	AUC (95% CI)	Sensitivity	Specificity	AUC (95% CI)	Sensitivity	Specificity	AUC (95% CI)	Sensitivity	Specificity	AUC (95% CI)
Reviewer 1	0.733	0.900	0.817 (0.661–0.972)	0.867	0.900	0.883 (0.756–1.000)	0.933	0.850	0.892 (0.773–1.000)	0.933	0.950	0.942 (0.849–1.000)
Reviewer 2	0.800	0.850	0.825 (0.675–0.975)	0.867	0.950	0.908 (0.792–1.000)	0.933	0.850	0.892 (0.773–1.000)	0.933	0.950	0.942 (0.849–1.000)

Meanwhile, there were no significant differences for the AiCE, AIDR 3D, and AIDR 3D–BBCT groups each other for two readers (*p* > 0.05).

### Vascular evaluation

**Fig. 4:**
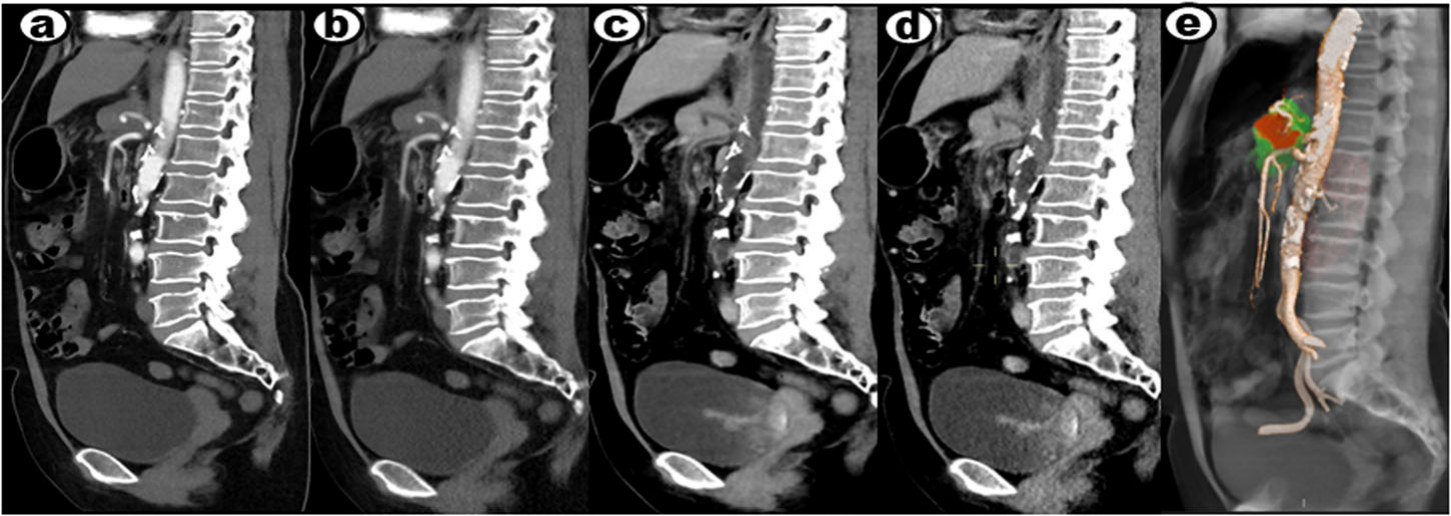
A 56-year-old woman was diagnosed with PDAC involving the body of the pancreas, with invasion of the CA and SMA. **a**–**d** Sagittal multi-planar reformation (MPR) CT angiographies were reconstructed using AiCE, AIDR 3D, AiCE–BBCT, and AIDR 3D–BBCT algorithms to visualize the arteries at the same window and center levels. The images allowed for clear visualization of the invaded CA (indicated by an arrow) in each reconstruction. **e** Additionally, a sagittal GIR fusion image obtained using the AiCE algorithm was generated. In this fusion image, the normal pancreatic parenchyma was represented in green, while the tumor was depicted in red. The invaded CA and SMA were well delineated, providing a comprehensive visualization of the tumor’s involvement with these vessels. The images were acquired with a slice thickness of 1 mm. The BBCT technique effectively reduced the enhanced CT value within the vascular lumen, resulting in better visualization of the vascular wall

**Fig. 5:**
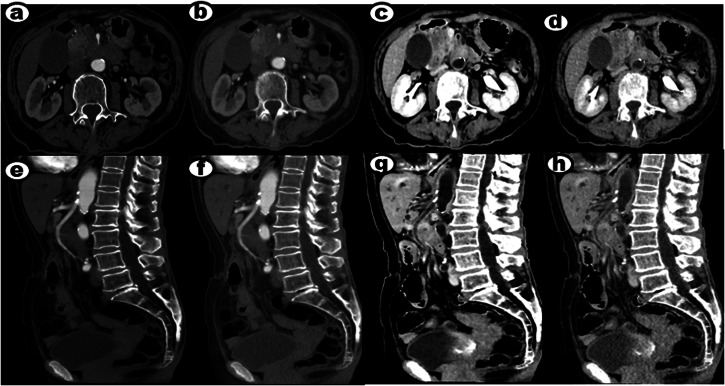
An 83-year-old woman was diagnosed with PDAC involving the head of the pancreas, with invasion of the SMA. **a**–**d** Axial arterial phase images were reconstructed using AiCE, AIDR 3D, AiCE–BBCT, and AIDR 3D–BBCT algorithms, providing detailed visualization of the tumor and the invaded SMA. The images demonstrated the presence of the tumor and the extent of vascular invasion. **e**–**h** Sagittal MPR CT angiographies were reconstructed at the same window and center levels using AiCE, AIDR 3D, AiCE–BBCT, and AIDR 3D–BBCT algorithms. These images further highlighted the invaded SMA, clearly delineating its involvement with the tumor. The slice thickness for image reconstruction was 1 mm. The BBCT technique effectively suppressed the enhanced CT value within the vascular lumen, resulting in improved visualization of the vascular wall. This allowed for a more accurate assessment of the invasion of the SMA by the PDAC

Finally, we performed a more detailed vascular evaluation of the 30 involved vessels in 12 patients, two of the cases are depicted in Figs. 4 and 5. The evaluated parameters included tumor contact of the vessel circumference (> 180 or ≤ 180), unilateral or bilateral stenosis, regular or irregular lumen, tumor invasion of blood vessel involvement length (> 1.2 cm or ≤ 1.2 cm), and the interclass correlation coefficient (ICC), which are summarized in Table 3. In the AiCE–BBCT group, all vascular evaluations between the two readers were classified as good to excellent (ICC: 0.861–0.935). Moreover, all vascular evaluations using AiCE–BBCT demonstrated good to excellent (ICC: 0.847–0.935). For the AIDR 3D group, all vascular evaluations were considered moderate (ICC: 0.652–0.744). However, when applying AiCE and AIDR 3D–BBCT groups, all vascular evaluations were rated as good (ICC: 0.775–0.871).

**Table 3:** The vascular evaluation of AIDR 3D, AIDR 3D–BBCT, AiCE, and AiCE–BBCT groups

Parameter		ICC (95% CI)	*p*-value
Tumor contact of the vessel circumference > 180° or ≤ 180°	AiCE–BBCTR1 and AiCE–BBCTR2	0.935 (0.870–0.969)	< 0.001
	AiCE–BBCTR1 and the gold standard	0.871 (0.780–0.928)	< 0.001
	AiCE and the gold standard	0.804 (0.628–0.901)	< 0.001
	AIRD 3D and the gold standard	0.740 (0.525–0.807)	< 0.001
	AIRD 3D–BBCT and the gold standard	0.804 (0.628–-0.901)	< 0.001
Unilateral or bilateral stenosis	AiCE–BBCTR1 and AiCE–BBCTR2	0.921 (0.835–0.958)	< 0.001
	AiCE–BBCTR1 and the gold standard	0.847 (0.704–0.924)	< 0.001
	AiCE and the gold standard	0.775 (0.580–0.886)	< 0.001
	AIRD 3D and the gold standard	0.707 (0.467–0.849)	< 0.001
	AIRD 3D–BBCT and the gold standard	0.775 (0.580–0.886)	< 0.001
Involvement length > 1.2 cm or ≤ 1.2 cm	AiCE–BBCTR1 and AiCE–BBCTR2	0.935 (0.870–0.967)	< 0.001
	AiCE–BBCTR1 and the gold standard	0.866 (0.738–0.934)	< 0.001
	AiCE and the gold standard	0.804 (0.628–0.901)	< 0.001
	AIRD 3D and the gold standard	0.652 (0.371–0.821)	< 0.001
	AIRD 3D–BBCT and the gold standard	0.800 (0.622–0.899)	< 0.001
Regular or irregular lumen	AiCE–BBCTR1 and AiCE–BBCTR2	0.861 (0.731–0.930)	< 0.001
	AiCE–BBCTR1 and the gold standard	0.935 (0.869–0.968)	< 0.001
	AiCE and the gold standard	0.800 (0.622–0.899)	< 0.001
	AIRD 3D and the gold standard	0.744 (0.520–0.871)	< 0.001
	AIRD 3D–BBCT and the gold standard	0.871 (0.747–0.936)	< 0.001

The gold standard for diagnosis is based on pathological findings or results from exploratory laparotomies

*CI* confidence interval

## Discussion

We aimed to assess the subjective image quality and diagnostic performance of AIDR 3D and AiCE combined with and without BBCT in abdominal contrast-enhanced CT examinations, specifically focusing on their ability to evaluate PDAC.

Our findings revealed that the AiCE and AiCE–BBCT groups exhibited superior image noise and diagnostic acceptability in both arterial and portal venous phases compared to the AIDR 3D and AIDR 3D–BBCT groups. We compared the image noise and diagnostic acceptability between the AIDR 3D and AIDR 3D–BBCT groups, and the AiCE and AiCE–BBCT groups, in either arterial or portal venous phases, but it was not statistically significant. Under the same radiation dose conditions, AiCE demonstrated better subjective image quality than AIDR 3D, which is consistent with previous studies [11, 12, 17]. The diagnostic acceptability in both arterial and portal venous phases was slightly better in the AIDR 3D–BBCT group compared to the AIDR 3D group, and in the AiCE–BBCT group compared to the AiCE group, but there were no significant differences (*p* > 0.05). We speculate that most PDACs exhibit limited enhancement in the arterial phase due to the reason that PDAC is a hypo-vascular tumor [22, 23]. This study did not involve the comparison of radiation doses, in terms of image quality, superior image noise referring to a higher diagnostic acceptability. However, BBCT has the potential to improve contrast and thereby enhance tumor detection. High-quality tumor detection plays a vital role in whether the tumor contacts blood vessels or not [24]. The subtraction technique has been extensively utilized in CT imaging. However, accurately highlighting vascular wall images on CT scans remains a significant challenge. BBCT, as a novel technique, aims to enhance contrast in retrospectively acquired enhanced CT images. Previous studies have explored the application of BBCT in carotid artery imaging [18]. In our study, we innovatively applied BBCT to abdominal contrast-enhanced CT scans for visualizing abdominal blood vessels.

Comparing the AiCE–BBCT group with the AIDR 3D and AIDR 3D–BBCT groups, we observed higher specificity and sensitivity in the diagnostic capability of vascular invasion in PDAC. In terms of the diagnostic capability of CT for vascular invasion in PDAC, specificity ranged from 82% to 100%, and sensitivity ranged from 70% to 96% [24]. We found the sensitivity and specificity of AiCE–BBCT was 93.3% and 95.0%, respectively. That indicated that AiCE–BBCT significantly improved the diagnostic capability of CT for detecting vascular invasion in PDAC.

In the guidelines, tumor contact ≤ 180 of the vessel circumference is referred to as “abutment,” while tumor contact > 180 is “encasement.” In the process of vascular reconstruction surgery, mapping the anatomy of individual vessels precisely is essential to help reduce postoperative complications [25]. The length of vessel resection has also been shown to correlate with long-term survival [4]. Irregularity of the vessel contour or changes in caliber are considered signs of vascular invasion [25]. Therefore, we evaluated the involved vessels accordingly and compared the consistency of four reconstruction methods with pathological findings or results from exploratory laparotomies. The evaluation using AiCE–BBCT showed better consistency compared to the AiCE, AIDR 3D, and AIDR 3D–BBCT groups. The interclass correlation coefficient (ICC) values ranged from 0.861 to 0.935, representing a perfect consistency between the two observers in the AiCE–BBCT group. The reason for the improved consistency may be attributed to BBCT, which highlights the visualization of the vascular wall [20]. Previous research [18] has shown that the CE-boost technique, which utilizes dark-blood imaging, significantly improves image quality and discernibility.

BBCT is also used to clearly display the carotid wall and accurately diagnose the rate of carotid artery stenosis and plaque load [20]. CE-boost technology is generally used by a large number of CT vendors and requires very little computing power, whereas AiCE–BBCT technology is more advanced and has great potential for assessing abdominal vascular invasion.

Sample size is a concept related to precision and statistical power [26]. So, a post-hoc power calculation of the study sample size was conducted to evaluate the actual effectiveness of detecting a specific effect with *α* error of 0.05 in the sample size of this study. Post-hoc power calculations supported strong power (more than 90%), in other words, the sample size of this study is reasonable, and the results are convincing.

There were several limitations in our study. Firstly, the present study had few samples in one center. To verify the validity and universality of the study, it is necessary to increase the sample size and re-count the statistics in the later study to reduce the error, and it is possible to conduct a joint multi-center prospective study to further confirm its research value. Secondly, the repeated addition of similar iodine images to the primitive image will possibly increase the motion artifact. That can result in blurred images, making it difficult to accurate images. Thirdly, due to the better image quality of AiCE–BBCT, we will further investigate low-dose imaging protocols to evaluate vascular invasion in PDAC patients.

In conclusion, the study findings suggest that the combination of the BBCT technique with AiCE improves both the image quality of PDAC images and the diagnostic accuracy for tumor vascular invasion. The improved image quality provided by BBCT contributes to better visualization of vascular involvement by the tumor. This has the potential to enhance the accuracy of diagnosing vascular invasion, which is a critical factor in determining treatment strategies and patient outcomes.

## Data Availability

The datasets are available from the corresponding author with a reasonable request.

## Abbreviations

AiCE: Advanced intelligent clear-IQ engine
AIDR 3D: Adaptive iterative dose reduction 3D
AUC: Area under the curve
BBCT: Black blood CT technique
CA: Celiac artery
CE-boost: Contrast-enhancement boost
CHA: Common hepatic artery
CT: Computed tomography
DLP: Dose-length product
DLR: Deep learning reconstruction
GIR: Global illumination rendering
Hybrid-IR: Hybrid-iterative reconstruction
ICC: Intraclass correlation coefficient
MPR: Multi-planar reformation
NCCN: National Comprehensive Cancer Network
NS: Normal saline
PDAC: Pancreatic ductal adenocarcinoma
PV: Portal vein
SD: Standard deviation
SMA: Superior mesenteric artery
SMV: Superior mesenteric vein

## References

[1] Mizrahi JD, Surana R, Valle JW, Shroff RT (2020) Pancreatic cancer. Lancet 395:2008–2020 32593337 10.1016/S0140-6736(20)30974-0

[2] Fowler KJ (2018) CT assessment of pancreatic cancer: What are the gaps in predicting surgical outcomes? Radiology 289:719–720 30251932 10.1148/radiol.2018181912

[3] Tempero MA, Malafa MP, Al-Hawary M et al (2021) Pancreatic adenocarcinoma, version 2.2021, NCCN clinical practice guidelines in oncology. J Natl Compr Canc Netw 19:439–457 33845462 10.6004/jnccn.2021.0017

[4] Zaky AM, Wolfgang CL, Weiss MJ, Javed AA, Fishman EK, Zaheer A (2017) Tumor-vessel relationships in pancreatic ductal adenocarcinoma at multidetector CT: different classification systems and their influence on treatment planning. Radiographics 37:93–112 27885893 10.1148/rg.2017160054

[5] Lao Y, David J, Fan Z et al (2020) Quantifying vascular invasion in pancreatic cancera contrast CT based method for surgical resectability evaluation. Phys Med Biol 65:105012 32187583 10.1088/1361-6560/ab8106 PMC7316342

[6] Hong SB, Lee SS, Kim JH et al (2018) Pancreatic cancer CT: prediction of resectability according to NCCN criteria. Radiology 289:710–718 30251929 10.1148/radiol.2018180628

[7] Zhou X, Xu D, Wang M et al (2023) Preoperative assessment of peripheral vascular invasion of pancreatic ductal adenocarcinoma based on high-resolution MRI. BMC Cancer 23:1092 37950223 10.1186/s12885-023-11451-8 PMC10638695

[8] Rigiroli F, Hoye J, Lerebours R et al (2021) CT radiomic features of superior mesenteric artery involvement in pancreatic ductal adenocarcinoma: a pilot study. Radiology 301:610–622 34491129 10.1148/radiol.2021210699 PMC9899097

[9] Kulkarni A, Carrion-Martinez I, Jiang NN et al (2020) Hypovascular pancreas head adenocarcinoma: CT texture analysis for assessment of resection margin status and high-risk features. Eur Radiol 30:2853–2860 31953662 10.1007/s00330-019-06583-0

[10] Jang JK, Byun JH, Kang JH et al (2021) CT-determined resectability of borderline resectable and unresectable pancreatic adenocarcinoma following FOLFIRINOX therapy. Eur Radiol 31:813–823 32845389 10.1007/s00330-020-07188-8

[11] Ludes G, Ohana M, Labani A, Meyer N, Molire S, Roy C (2023) Impact of a reduced iodine load with deep learning reconstruction on abdominal MDCT. Medicine (Baltimore) 102:e34579 37657067 10.1097/MD.0000000000034579 PMC10476859

[12] Nagayama Y, Iwashita K, Maruyama N et al (2023) Deep learning-based reconstruction can improve the image quality of low radiation dose head CT. Eur Radiol 33:3253–3265 36973431 10.1007/s00330-023-09559-3

[13] Arndt C, Guttler F, Heinrich A, Burckenmeyer F, Diamantis I, Teichgraber U (2021) Deep learning CT image reconstruction in clinical practice. Rofo 193:252–261 33302311 10.1055/a-1248-2556

[14] Tatsugami F, Higaki T, Nakamura Y et al (2019) Deep learningbased image restoration algorithm for coronary CT angiography. Eur Radiol 29:5322–5329 30963270 10.1007/s00330-019-06183-y

[15] Yi Y, Xu C, Xu M et al (2021) Diagnostic improvements of deep learning-based image reconstruction for assessing calcification-related obstructive coronary artery disease. Fronti Cardiovasc Med 8:758793 10.3389/fcvm.2021.758793 PMC8595262 34805313

[16] Xu J, Wang S, Wang X et al (2022) Effects of contrast enhancement boost postprocessing technique in combination with different reconstruction algorithms on the image quality of abdominal CT angiography. Eur J Radiol 154:110388 35714492 10.1016/j.ejrad.2022.110388

[17] Tamura A, Mukaida E, Ota Y, Nakamura I, Arakita K, Yoshioka K (2022) Deep learning reconstruction allows low-dose imaging while maintaining image quality: comparison of deep learning reconstruction and hybrid iterative reconstruction in contrast-enhanced abdominal CT. Quant Imag Med and Surg 12:2977–2984 10.21037/qims-21-1216 PMC9014148 35502368

[18] Li J, Zhang Y, Hou J et al (2023) Clinical application of dark-blood imaging in head and neck CT angiography: effect on image quality and plaque visibility. Acad Radiol 31:2478–2487 10.1016/j.acra.2023.11.015 38042623

[19] Grob D, Smit E, Prince J et al(2019) Iodine maps from subtraction CT or dual-energy CT to detect pulmonary emboli with CT angiography: a multiple-observer study. Radiology 292:197–205 31084482 10.1148/radiol.2019182666

[20] Lu Y, Cao R, Jiao S et al (2024) A novel method of carotid artery wall imaging: black-blood CT. Eur Radiol 34:2407–2415 37736805 10.1007/s00330-023-10247-5 PMC10957584

[21] Noda Y, Takai Y, Asano M et al (2023) Comparison of image quality and pancreatic ductal adenocarcinoma conspicuity between the low-kVp and dual-energy CT reconstructed with deep-learning image reconstruction algorithm. Eur J Radiol 159:110685 36603479 10.1016/j.ejrad.2022.110685

[22] Mathy RM, Fritz F, Mayer P et al (2021) Iodine concentration and tissue attenuation in dual-energy contrast-enhanced CT as a potential quantitative parameter in early detection of local pancreatic carcinoma recurrence after surgical resection. Eur J Radiol 143:109944 34482176 10.1016/j.ejrad.2021.109944

[23] Schawkat K, Manning MA, Glickman JN, Mortele KJ (2020) Pancreatic ductal adenocarcinoma and its variants: pearls and perils. Radiographics 40:1219–1239 32678699 10.1148/rg.2020190184

[24] Zins M, Matos C, Cassinotto C (2018) Pancreatic adenocarcinoma staging in the era of preoperative chemotherapy and radiation therapy. Radiology 287:374–390 29668413 10.1148/radiol.2018171670

[25] Al-Hawary MM, Francis IR, Chari ST et al (2014) Pancreatic ductal adenocarcinoma radiology reporting template: consensus statement of the Society of Abdominal Radiology and the American Pancreatic Association1. Gastroenterology 146:291–304 24355035 10.1053/j.gastro.2013.11.004

[26] Monti CB, Ambrogi F, Sardanelli F (2024) Sample size calculation for data reliability and diagnostic performance: a go-to review. Eur Radiol Exp 8:79 38965128 10.1186/s41747-024-00474-w PMC11224179

